# Use of Cumulative Incidence of Novel Influenza A/H1N1 in Foreign Travelers to Estimate Lower Bounds on Cumulative Incidence in Mexico

**DOI:** 10.1371/journal.pone.0006895

**Published:** 2009-09-09

**Authors:** Marc Lipsitch, Martin Lajous, Justin J. O'Hagan, Ted Cohen, Joel C. Miller, Edward Goldstein, Leon Danon, Jacco Wallinga, Steven Riley, Scott F. Dowell, Carrie Reed, Meg McCarron

**Affiliations:** 1 Department of Immunology and Infectious Diseases, Harvard School of Public Health, Boston, Massachusetts, United States of America; 2 Center for Population Health Research, National Institute of Public Health, Cuernavaca, Mexico; 3 Department of Epidemiology, Harvard School of Public Health, Boston, Massachusetts, United States of America; 4 Division of Global Health Equity, Brigham and Women's Hospital, Harvard School of Public Health, Boston, Massachusetts, United States of America; 5 Fogarty International Center, National Institutes of Health, Bethesda, Maryland, United States of America; 6 Department of Biological Sciences, University of Warwick, Coventry, United Kingdom; 7 Epidemiology and Surveillance Unit, Centre for Infectious Disease Control, National Institute of Public Health and the Environment (RIVM), Bilthoven, The Netherlands; 8 School of Public Health and Department of Community Medicine, University of Hong Kong, Hong Kong, People's Republic of China; 9 Division of Global Disease Detection and Emergency Response, Centers for Disease Control and Prevention, Atlanta, Georgia, United States of America; 10 Influenza Division, National Center for Immunization and Respiratory Diseases, Centers for Disease Control and Prevention, Atlanta, Georgia, United States of America; Yale University, United States of America

## Abstract

**Background:**

An accurate estimate of the total number of cases and severity of illness of an emerging infectious disease is required both to define the burden of the epidemic and to determine the severity of disease. When a novel pathogen first appears, affected individuals with severe symptoms are more likely to be diagnosed. Accordingly, the total number of cases will be underestimated and disease severity overestimated. This problem is manifest in the current epidemic of novel influenza A/H1N1.

**Methods and Results:**

We used a simple approach to leverage measures of incident influenza A/H1N1 among a relatively small and well observed group of US, UK, Spanish and Canadian travelers who had visited Mexico to estimate the incidence among a much larger and less well surveyed population of Mexican residents. We estimate that a minimum of 113,000 to 375,000 cases of novel influenza A/H1N1 have occurred in Mexicans during the month of April, 2009. Such an estimate serves as a lower bound because it does not account for underreporting of cases in travelers or for nonrandom mixing between Mexican residents and visitors, which together could increase the estimates by more than an order of magnitude.

**Conclusions:**

We find that the number of cases in Mexican residents may exceed the number of confirmed cases by two to three orders of magnitude. While the extent of disease spread is greater than previously appreciated, our estimate suggests that severe disease is uncommon since the total number of cases is likely to be much larger than those of confirmed cases.

## Introduction

A reliable estimate of the cumulative number of infections for an emerging disease, such as novel influenza A/H1N1, is critical to determine both the magnitude of the problem and the severity of disease. Cumulative incidence is the most direct estimate of the magnitude of the epidemic, while cumulative deaths and hospitalizations must be divided by cumulative incidence (with appropriate correction for reporting delays and censoring [1]) to estimate the probability of severe outcomes for individuals that become infected. While critical for situational awareness, cumulative incidence is often difficult to measure in a large epidemic, because often there is a bias toward ascertainment of severe cases.

Where underreporting of asymptomatic and mild cases, especially those that do not present for medical care, is likely, there is a need for nonstandard approaches to estimate the magnitude of the epidemic and severity of disease. Here we propose and apply such a method to estimate the number of cases of novel influenza A/H1N1 in Mexico up to approximately April 30, 2009, based on the number of cases observed in foreign travelers. Intuitively, the notion is that such travelers act as “canaries in the mine” who briefly experience the daily risk of infection prevalent in Mexico during their visit, then return home to areas where, given the elevated level of concern, they may be detected as cases of novel H1N1, even if not severe. By assuming (conservatively) that the risk of infection experienced by Mexicans is at least equal to that experienced by visitors, and using travel data to assess the amount of person-time at risk for visitors, we estimate the incidence rate in proportion to the Mexican population, and estimate a lower-bound of how many cases may have been present in Mexico at a defined time.

Here we estimate that at least 113,000–375,000 cases of novel H1N1 influenza occurred in Mexicans before the end of April, 2009. We discuss the uncertainties associated with this estimate and present our rationale for why this number represents a lower bound for the true number. Finally, we discuss the implications for estimating the case-fatality proportion of this infection in Mexico.

## Results

### Baseline estimate

We estimate that approximately 375,000 Mexicans were infected with novel H1N1 influenza with symptom onset up to approximately April 30, 2009. This estimate derives from 283 cases among US, UK, Spanish and Canadian travelers, counting confirmed and probable cases for the US and confirmed cases only for the other two countries. Citizens of these countries together accounted for approximately 689,250 airplane passenger visits to Mexico in the period April 1–30, 2009, and international visitors to Mexico had a mean length of stay of approximately 3.5 days, for a total of 2.4 million person-days of exposure during this period (Table 1). This implies that visitors experienced an incidence rate of 91 cases per million person-days at risk. In the same period, the Mexican population of approximately 107 million persons had 30×107 million, or 3.2 billion person-days of exposure.

**Table 1:** Cases of novel influenza A/H1N1 among travelers to Mexico from three countries as of May 6, 2009 (Canada) or May 8, 2009 (US, UK, Spain) and associated estimates.

	US (confirmed+probable)	Canada (confirmed)	UK (confirmed)	Spain (confirmed)	Total
Cases with Mexico travel history	132	62	19	70	283
Cases with travel history known/total cases	928/1890	86/179	37/38	93/93	
With only one case per possible cluster, and near border cases removed	85	56	17	no data to assess clusters; 70 assumed	228
Travel volume for April	526,861	119,473	22,013	20,903	668,347
Inferred incidence rate (/million person-days)	72	148	246	957	117
Inferred cases in Mexico	229,000	475,000	789,000	3,062,000	375,000
Inferred incidence rate (/million person-days)*	18	44	55	241	35
Inferred cases in Mexico*	*59,000*	*142,000*	*178,000*	*771,000*	*113,000*

### Sensitivity analysis: unknown travel history

Travel history was known for 49% (929/1890) of US confirmed cases and 48% (86/179) of Canadian confirmed cases, 97% (37/38) of the UK cases and 100% (93/93) of Spanish cases. If the proportion of cases with travel history to Mexico is assumed to be the same for those with missing data in this field, the imputed number of total cases with travel history would rise to 418, and the implied number of cases in Mexicans would rise to 554,000. We strongly suspect that travel history is more likely to be known in those who did travel to Mexico than in those who did not, which would suggest that the correction for missing travel history should be somewhat less than assumed here. We therefore do not include this large estimate in our overall range of estimates.

### Sensitivity analysis: possible clusters among travelers, and border state cases

Several cases among travelers may have resulted from clusters of exposure and/or from transmission within the traveling group. In order to exclude the effects of transmission among travelers or cases of disease imported by means other than air travel, we provide a revised estimate calculated from a subset of 228 cases. This reduced number of cases excludes both secondary cases within putative clusters of travelers (these data were available for travelers from each country except Spain) and excludes US cases residing in or south of the closest major city to the Mexican border who may have visited by means other than air travel. This approach yields an estimate of 302,000 cases in Mexicans; additional correction for clustering in Spanish cases, if the required data were available, would further reduce this figure.

### Sensitivity analysis: length of stay

For reasons discussed below, we believe that 3.5 days is an appropriate estimate for the mean duration of stay in Mexico for all visitors, which heavily weights US visitors because the US is the largest source of visitors. However, given that one study suggests a considerably longer length of stay [2], and that non-US visitors likely stay longer given the longer trip involved, we performed a sensitivity analysis assuming that visitors from the US, Canada and European countries have lengths of stay of 8.7, 10.5, and 13.9 days respectively, using numbers from an unpublished 2008 update of the 2001–5 survey (Gerardo Vazquez, Mexico Ministry of Tourism, personal communication). Using the data with possible clusters and near-border cases removed, produces a low estimate of 113,000 cases in Mexican residents.

### Sensitivity analysis: non-homogeneous disease across Mexico

This analysis assumes that incidence during April was homogeneous across 107 million Mexicans. If the rates of disease among Mexicans in travel destinations was higher or lower than elsewhere, this might substantially alter these estimates. The national cumulative incidence of suspect cases as of May 9 was 17.32/100,000, which was 16x higher than that in Puebla, the state with the lowest incidence, and 4x lower than that in Distrito Federal, the capital, with the highest reported incidence. Quintana Roo, the state containing Cancun, which is the most popular single destination for travelers from these countries, reported incidence of 12.10/100,000. If these incidence numbers reflect true incidence variation in the country (which is unlikely to be the only source of variation), then total Mexican incidence should be 1.4 times higher than that estimated from Cancun travelers, or 4x lower than that estimated for Mexico City travelers. Unfortunately, destination data are not available for the majority of travel-associated cases in any of the four countries we considered.

## Discussion

We have estimated that there are likely to have been at least 113,000–375,000 cases of novel H1N1 influenza among Mexicans with onset during the month of April, 2009. Taking into account what we consider to be extreme sensitivity analyses, this estimate could change by approximately 2-fold in either direction. This exceeds the number of confirmed cases reported to WHO, 1204 as of May 8, 2009 (http://www.who.int/csr/don/GlobalSubnationalMaster_20090508_1815.jpg), by a factor of approximately 100 or more.

It is unsurprising that we estimate a larger number than the number of cases confirmed in Mexico, since ascertainment there has been particularly focused on severe cases. Nevertheless, we regard this estimate as likely a lower bound on the actual number of cases in Mexico, for two principal reasons. First, the analytic approach assumes that the incidence rate in Mexicans in Mexico is equal to that in travelers. If indeed the infection has been transmitting extensively within Mexico, one would expect that the exposure of travelers to the virus would be somewhat less than that of residents, due to nonrandom mixing between residents and travelers; travelers should be less exposed to residents than other residents are. Prior models of influenza transmission (set in the United States) have assumed that 36–51% of influenza transmission takes place outside of home or school [3]. One might roughly estimate that this is the proportion of transmission to which both visitors and residents would be exposed, suggesting that incidence in residents might be 2–3x as high as that in visitors; however, this approach has obvious limitations given the uncertainty of those estimates and the fact that they were made for a different country.

Second, while most cases ascertained in the traveler population to date have been mild, one nonetheless expects that many mild cases (as well as probable but unconfirmed cases) in travelers are absent from our calculations. A survey in New York City, where case ascertainment was aggressive surrounding the St. Francis School outbreak, indicated that over 1000 persons associated with the school experienced influenza-like illness, in a period where only 74 confirmed or probable cases were ascertained. If these figures reflect the typical rate of under-reporting in the United States, then the inferred figures from Mexico should increase by >1000/74 = 14-fold. Likewise, any foreign residents who became ill in Mexico (rather than in their home country) may have been missed in our counts of travelers. In essence, the method used here is a way to estimate cases in a population where they are likely being undercounted, based on travelers to countries in which undercounting, though present, is less severe. Since the inferred number of cases in Mexican residents scales linearly with the number observed in travelers, the number in Mexican residents is likely to be considerably higher than we have estimated.

Forty-eight deaths were observed up to May 9 among laboratory-confirmed cases in Mexico [4]. While it might be tempting to calculate a case-fatality proportion by dividing this number by the estimated number of cases in Mexico, such a calculation would likely be misleading, for several reasons. In a growing epidemic, given a significant delay from illness onset to death [5], one expects to underestimate the case-fatality proportion as the deaths reflect cases from an earlier, smaller phase of the epidemic [6]. Also, counting only laboratory confirmed deaths is likely to result in a significant underestimation of the true number of deaths, because of insensitivity depending on the timing and adequacy of the specimen, the fact that many severe pneumonia patients were not tested (approximately 1000–2000 such cases typically occur in Mexico in April [7]), and the fact that a majority of influenza deaths are attributed to circulatory causes rather than identified as pneumonia or influenza [8]. Nonetheless, as the number of deaths accumulates, especially if illness onset dates are available for fatal cases, our estimates may provide an appropriate denominator for revised estimates of the case-fatality proportion. The number of hospitalizations associated with suspect cases was 6,754 as of May 9 [4], which combines with our denominator to give a hospitalization proportion of about 2%, closer to figures observed elsewhere.

We have shown in Table 1 the estimates obtained using only travelers from each country individually. Here, the US-based estimates are the lowest, with greater estimates from those based on Canadians and still greater estimates based on Europeans. In part this may reflect a longer duration of trips for travelers from more distant destinations, but even using the destination-specific duration data does not remove this effect. As we note below, we cannot rule out the possibility that some transmission occurred on airplanes; such transmission might be more likely in travelers flying longer distances. Differences in patterns of exposure within Mexico, chance variation and other factors must account for the remaining differences.

This simple model has several principal limitations. First, we do not incorporate exposure of travelers who arrive by ship or overland, only by air. While we have excluded from the numerator the one traveler case with a known cruise ship exposure, we may have slightly overestimated the incidence in travelers by neglecting such exposures. Second, our calculations make the assumption that incidence is uniform geographically throughout Mexico and across age group. All but one state in Mexico have now reported cases (http://portal.salud.gob.mx/sites/salud/descargas/pdf/influenza/situacion_actual070511.pdf), and all have at least suspect cases [3], so it is likely reasonable to assume that persons throughout Mexico were exposed to some extent. However, the exposure may not have been uniform. This may be a further reason to consider our estimate as a lower bound, since the detected cases are heavily concentrated in the State of Mexico and the Distrito Federal, the destination of <18% of visitors from these countries, while the most popular airport of entry for visitors from the US, UK and Canada in April 2009 was Cancun, which accounted for 47.5%–74.5% of visitors for each nationality but had relatively low reported incidence. As the pandemic has evolved, it has become clear that different age groups experience different risks of confirmed and probable infection with the pandemic virus, with the highest rates of confirmed and probable infection among persons under 25 years old (http://www.cdc.gov/h1n1flu/surveillanceqa.htm). Finally, we assume that transmission to travelers occurred in Mexico, not on an aircraft. An influenza outbreak on an aircraft has been documented [9], and if a cluster of such infections were included in our numbers, it would result in an overestimate of incidence in Mexico. Notably, 36% of travel-associated cases in Spain for whom data were available were symptomatic during the inbound flight; given the incubation period of influenza, these travelers, at least, could not plausibly have become infected during the flight [10].

Our estimates of cases are larger, by about 10-fold, than those reported by Fraser et al. [11]. Importantly, this reflects the fact that we base ascertainment on numbers available on May 6–8, while Fraser et al. base ascertainment on numbers available on April 30. With rapid epidemic growth, the difference of one week is likely to account for a difference of perhaps 2-8-fold. Also, Fraser et al. use a longer mean length of stay (9 days) and a larger travel volume. Estimates of the length of stay cited by Fraser et al. [11] were close to 9 days in 2001-5 [2], and we have considered a sensitivity analysis based on an updated version of that survey, using numbers specific to origin of the travelers. For our primary analysis, however, we used figures from the Ministry of Tourism indicating a mean length of stay of 3.4 days (see Methods), while an independent study conducted by the National Association of Hotels and Motels finds a similar value of 3.6 days for the mean length of hotel stay by foreign visitors, and a very recent survey found that the majority of US leisure travelers interested in visiting Mexico take vacations for 4 nights or less (personal communication). Our travel volumes are lower in part because we have used citizenship rather than first destination outside Mexico (to better reflect likely final destination) and have used data on number of incoming passengers (corrected to estimate outgoing passengers) rather than flight data, which may perhaps reflect capacities rather than actual numbers. Altogether, these differences in data sources could account for approximately a 3-fold variation in estimates, apart from the variation due to different time periods considered.

Accurate estimation of the magnitude of an emerging epidemic is essential for maintaining situational awareness and determining a rational public health response. The simple approach applied here indicates that the likely number of cases of H1N1 influenza among Mexican residents during the month of April, 2009 was at least two orders of magnitude larger than that detected. While such calculations should not be interpreted as precise estimates of cumulative incidence, they provide important perspective in interpreting data from detected cases in situations where extensive surveillance is unlikely to occur.

## Methods

### Data sources

#### Cases in travelers

Cases ascertained in the US in travelers were obtained from the US CDC line list dated May 8 at 0100 EDT, reflecting cases reported up to May 7. Possible clusters of traveler cases were detected by manual scan of the line list for cases with common county of report, closely related onset dates, and no indication that they lived in different households. Cases ascertained in Canada in travelers were obtained from a copy of the Canadian line list dated May 6 residing at the US CDC. Possible clusters of traveler cases were noted on the line list itself. Cases ascertained in the UK in travelers were obtained from a comprehensive scan of press reports cross-checked with UK Health Protection Agency daily updates to ensure consistency of numbers, and possible clusters were ascertained the same way. One case from the United States known to be in a woman visiting Mexico on a cruise ship was excluded since cruise ship visitors were not included in our travel estimates. The number of cases in travelers was denoted 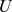. Use of line lists from 6–8 days after our period of interest was selected because for those entering the US CDC line list, the mean delay from symptom onset was 7 days. Hence, the US data, which represented the majority of cases, should be representative of cases with onset in the period up to April 30. The number of cases from Spain was taken from the recent report produced by the Surveillance Group in Spain [10].

### Person-time at risk

The Mexican population was assumed to be 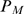 = 106,682,518 persons as estimated by the National Council for Population of Mexico http://www.conapo.gob.mx/index.php?option=com_content&view=article&id=125&Itemid=193. Estimates of the number of travelers returning from Mexico during the period April 1–30 were obtained using data from Mexican immigration records deposited in the Sistema Integral de Operación Migratoria (SIOM). This database contains information on the citizenship of all travelers arriving into Mexican airports. Assuming that the populations of inbound and outbound travelers from Mexico are in near-steady state the number of inbound travelers should give a reliable estimate of the number of outbound travelers. Records were abstracted for the period April 1–30. Note that our method is not strongly sensitive to the exact period considered, since additional days would proportionately increase the person-time for Mexican residents and approximately proportionately increase the person-time for visitors. We did not decrement the person-time to account for time no longer at risk once a Mexican resident was infected.

The number of Canadian, British and Spanish travelers arriving into Mexico began to drop off on April 27th, likely in response to the media coverage of the outbreak, while the number of US travelers to Mexico began to decrease on April 26th. As it is unlikely that the number of outbound travelers decreased over this period we calculated the average number of travelers arriving into Mexico for each day of the week using data for the first three weeks of April. These estimates were used instead of the actual daily numbers of travelers for the latter days of April. The total number of travelers into Mexico was denoted 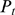. The mean duration of stay was assumed to be 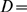
3.5 days. This was based on a mean stay of 3.6 days from survey data for hotel stays in April 2009 from the National Association of Hotels and Motels of Mexico (personal communication) and on a mean stay of 3.4 days from survey data posted by the Mexican Tourism Ministry (http://www.sectur.gob.mx/wb/secturing/sect_8978_study_of__tourist_pr). In addition, a survey of a representative sample of US leisure travelers interested in visiting Mexico conducted in February and March of 2009 found that 74% of all vacations taken by this group were 4 nights or less (P. Yesawich, National Leisure Travel Monitor, personal communication).

Alternative estimates obtained from a 2008 Bank of Mexico tourism survey (Gerardo Vazquez, Mexico Ministry of Tourism, personal communication) an earlier version of which was used by Fraser et al. [11] give longer durations of stay overall and indicate heterogeneity by nationality in length of stay: 8.7 nights for US citizens, 10.5 nights for Canadians and 13.9 nights for others. These estimates were used in a sensitivity analysis. We note that with a typical incubation period of about 1–2 days for influenza A [13], individuals infected early on in a stay of two weeks would have been sick for a week or more before returning home, at which point they might have stopped shedding detectable virus. Our estimates are based on infections confirmed in the country to which a traveler returned, and would therefore tend to miss many such infections, suggesting that only a fraction of such a long stay would be “at risk” for the event of infection detected upon return.

### Analysis

If the incidence rate in Mexicans were 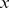 times that in visitors, then the following equality should hold, relating the incidence rate in each population: 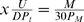, where in the month of April each Mexican had 30 days at risk, and each visitor had 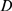 days at risk on average. Estimates for each quantity except for 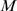, the unknown number of incident cases in Mexican residents, were provided from data, under the conservative assumption that 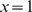, and the equation was solved for 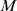. The major statistical uncertainty in our estimates comes from the number of visitors who were infected, which as a count with a value of 283 should have a coefficient of variation of 6%, negligible compared to the uncertainties of underreporting and differences in exposure of the visitor and resident populations. For this reason, statistical uncertainty was not explicitly quantified in our estimates.
